# Measuring woman’s perception of support and control during childbirth: Psychometric proprieties of the SCIB scale in an Italian sample

**DOI:** 10.1016/j.eurox.2025.100424

**Published:** 2025-08-22

**Authors:** Sara Molgora, Federica Bonazza, Maurizio Barbieri Carones, Enrico Maria Ferrazzi, Elizabeth Ford

**Affiliations:** a Department of Psychology, Catholic University of Milan, Largo Gemelli 1, Milan 20123, Italy; b Family Studies and Research University Centre, Catholic University of Milan, Largo Gemelli 1, Milan 20123, Italy; c Fondazione IRCCS Cà Granda, Ospedale Maggiore Policlinico, Milan 20122, Italy; d Department of Clinical Science and Community Health, Università degli Studi di Milano, Milan 20122, Italy; e Department of Primary Care and Public Health, Brighton and Sussex Medical School, Watson Building, Village Way, Falmer, Brighton BN1 9PH, UK

**Keywords:** Childbirth, Support, Control, Reliability, Validity

## Abstract

**Objective:**

This study aims to develop an Italian version of the Support and Control in Birth (SCIB) and to test its reliability and validity.

**Methods:**

The sample included 414 post-partum women, who were recruited 24/48 h after giving birth. A study-specific online questionnaire was designed to collect sociodemographic information and administer the translated Italian version of the SCIB. The construct validity of the Italian SCIB was first analyzed using Confirmatory Factor Analysis (CFA). Based on metrics showing a poor fit, Exploratory Factor Analysis (EFA) was then executed. The reliability of the instrument was verified through Cronbach’s alpha for each subscale and the total scale and test-retest correlation coefficient.

**Results:**

CFA was performed to test the original three-factor theoretical model; however, the CFA solution showed a poor fit. Specifically, χ2 = 2100, df= 492, p < 0.001, RMSEA = 0.0888 (0.085 – 0.0928), and CFI = 0.45. The theoretical model did not adequately fit the observed data. Thus, EFA was conducted; it yielded a six-factor structure. The six factors were named: Support: healthcare professionals’ guidance; Support: healthcare professionals’ presence; External control: control over medical procedures; External control: control over the informational process; Internal control: control over emotional and physical reactions; Internal control: control over pain. These factors replicate the originals dimensions, each divided into two further subfactors.

**Conclusion:**

Findings enhance knowledge about childbirth by cross-culturally adapting an instrument to assess perceptions of control and support, fundamental factors in birth experience. SCIB could help health professionals to monitor women's childbirth experiences and introduce tailored interventions.

## Introduction

Childbirth represents a crucial event within the process of transition to parenthood and its subjective experience can significantly impact on woman’s postpartum wellbeing as well as on her relationship with the baby and its development [1], [2], [3]. Indeed, a negative, even traumatic, experience is considered an important risk factor for the development of several forms of postpartum psychological diseases (e.g., anxiety symptoms, depressive symptoms, PTSD symptoms, etc.) and it is also associated with bad long-term outcomes in terms of sexual satisfaction, breastfeeding practice, and subsequent births [4].

The perceived quality of this experience is influenced by medical, obstetrical, psychological, and relational variables [5]. For example, obstetric emergencies (e.g., episiotomy) and/or fetal and neonatal complications have been associated with a more stressful birth experience [6], whereas findings on other obstetric factors (e.g., epidural analgesia and mode of delivery) are still inconclusive [7].

As for psychological variables, for example, high levels of perceived control and self-efficacy during labor and delivery (e.g., feeling capable of managing pain, having a sense of control over one’s body, having a sense of control on the birth environment, etc.) were found to be associated with higher levels of satisfaction and an overall better experience of birth [7;8], leading to a higher degree of self-esteem and an overall positive self-concept [9].

As for relational variables, high levels of perceived intrapartum support from the partner and from healthcare professionals (e.g., good communication, intrapartum care provided by non-pharmacological pain relief measures, involvement in the decision-making process, etc.) have been associated with a more positive experience of labor and delivery [7; 9–11). In contrast, ineffective shared decision-making is a significant predictive factor of a poor birth experience and the development of postpartum psychological problems [12]. Chabbert and colleagues [7], analyzing the risk factors of a negative childbirth experience, reported that the absence of the partner and low levels of perceived sense of control increased the explained variance from 36 % to 69 %, representing the most significant predictors. Furthermore, a significant association between labor and delivery perceived support and control emerged in several studies [9].

These psychological and relational variables seem to impact not only subjective experience of labor and delivery, but also their medical aspects. For example, women who had a greater sense of control reported lower levels of pain during childbirth and women who received intrapartum support had shorter labors [9]. These data suggest the importance of focusing specifically on the period of labor and delivery, analyzing its quality and the impacting factors.

The growing interest in labor and delivery perceived quality has led in recent years to the development of some instruments that aim to assess this experience, analyzing different dimensions. Among the most frequently cited and used tools, the Wijma Delivery Expectancy/Experience Questionnaire (W-DEQ) was developed to assess fear of childbirth both during pregnancy and after delivery, on the assumption that fear is the key dimension of this experience [13]. The Birth Satisfaction Scale-Revised (BSS-R) is a brief questionnaire designed to investigate women’s satisfaction with their birth experience, focusing on three dimensions (quality of care, personal attributes, and stress experienced) each assessed by only a few items [14]. The Birth Experience Questionnaire (BEQ) is a short questionnaire that evaluates several aspects (e.g., stress, fear, pain, support, control) of partners’ perception of the birth experience, with each aspect measured by a small number of items [15]. The Childbirth Experience Questionnaire (CEQ) is an extensive measure of women’s perceptions of labor and birth based on four dimensions (own capacity, professional support, perceived safety and participation) but it applies only to first-time mothers [16].

Ford and colleagues [17] developed the Support and Control in Birth (SCIB) scale. This 33-item scale aims to measure women’s perception of support and control (both internal and external), as multidimensional constructs, specifically focused on labor and birth and without overlapping with broader constructs [17]. The original version of the scale is composed of three factors: internal control (10 items; 1–10), external control (11 items; 11–21) and support (12 items; 22–33), accounting for 55 % of the variance and having good reliability. The authors claimed that the SCIB provides a reliable and comprehensive measure of support and control in birth. The scale considers both intrapersonal and interpersonal dimensions and includes a larger number of items. It was designed for use with both primiparous and multiparous women, thereby addressing the limitation of other instruments or integrating their strengths.

The SCIB scale was also tested and validated among Chinese, Turkish and Persian women [9], [18], [19]. Overall, these three studies confirm the structure of SCIB and report acceptable results in terms of reliability and validity; the Persian version of the scale showed suitable metrics for 31 items (items 14 and 17 were removed). To date, there are no studies that have investigated the psychometric proprieties of this instrument in the Italian context. The aim of the present study was to develop an Italian version of the SCIB and to test its reliability and validity.

## Materials and methods

### Procedure and participants

This cross-sectional study included a sample of Italian women. Participants were recruited 24/48 h after giving birth at a large university-based hospital using a convenience non-probabilistic sampling approach. Data were collected between May 2023 and October 2024. The inclusion criteria were as follows: 1) women without serious complications during pregnancy and labor (e.g., the death of the fetus or infant) hospitalized in the postnatal ward; 2) being above 18 years old; and 3) being able to speak, read, and understand the Italian language. Women with a current diagnosis of serious mental illness were excluded from this study. Midwives and nurses identified the women who met the inclusion criteria and invited them to participate. All participants were asked to sign a written consent form and complete an anonymous online questionnaire on the Qualtrics platform. The goals and procedures of the study were explained in the information sheet shown at the beginning of the questionnaire. Privacy and confidentiality regarding participants’ identities were guaranteed.

The minimum sample size was 330 women and was defined based on criteria that suggests a sample size 10 times the number of variables in the tool being validated and exceeding 300 participants [20], [21]. Of the 464 questionnaires collected, 50 were incomplete (i.e., with a response rate of less than 80 %), leaving 414 fully completed questionnaires comprising the full sample.

To check repeatability, a one-month test-retest was performed. After one month, the researchers sent an email to participants who had agreed to share their contact details and asked them to complete the SCIB through a Qualtrics link. Participants who did not respond to the initial request received reminders after one week. At the end of each online survey, participants were asked to enter an alphanumeric code consisting of the first letter of the name and the last name, date of birth, and the last two digits of their telephone number. The alphanumeric code made it possible to match the data shared by the participants in the two assessments. The retest sample consisted of 86 participants.

### Materials

#### Support and control in birth (SCIB)

The SCIB is a self-report questionnaire that assesses perceived support and control of women during childbirth. This scale comprises 33 items rated on a five-point Likert scale (1 =completely agree; 5 = completely disagree). Items are organized in three dimensions: Internal control (10 items, including “I was in control of my emotions”), External control (11 items, including “I could influence which procedures were carried out”), and Support (12 item, including “The staff encouraged me to try new ways of coping”). The total score ranges from 0 to 165. For each subscale, higher scores indicate greater control or support. To develop the Italian version of the SCIB, we followed University of North Dakota’s guidelines. The goal of translating the SCIB into Italian was to produce a conceptually identical version of the tool that the patients could easily comprehend. First, a qualified translator translated the English version of the SCIB into Italian. To agree with the SCIB scale, the translator and the researchers talked about each item. The Italian version was then translated back into English by another translator. The Italian version was refined by comparing the original and translated versions.

#### Sociodemographic and clinical information

A study-specific questionnaire was designed to sociodemographic information, such as gender, age, level of education, relationship duration. Clinical information about the current pregnancy, such as the type of conception, presence and type of complications, was collected by one researcher of this study.

### Data analysis

Descriptive statistics were performed to describe variables of the background form. Specifically, mean ± standard deviation for continuous variables, frequency and percentages for categorical variables were calculated.

#### Validity

The construct validity was first analyzed using Confirmatory Factor Analysis (CFA). CFA is a structural equation model that assesses the validity of latent constructs by examining the relationships between observed variables and a hypothesized theoretical factor model [22], [23]. The validity analysis reassesses the original three-factor model proposed by Ford and colleagues [17]. The original authors first conducted a qualitative study, which revealed three fundamental control-related themes that formed the basis of the three-dimensional model, which was then confirmed by principal component analysis. The three-factor model includes 1) internal control, 2) external control, and 3) support. This model has already been analyzed in previous studies, for example, the study by Liu and colleagues [9], who assessed the validity of the Chinese version of the SCIB using CFA.

Hence, we executed CFA to evaluate the extent to which this theoretical measurement model of SCIB was replicated in the Italian sample. After preliminary checks, CFA was performed with Maximum Likelihood estimation method. To evaluate model fit, the χ2 value, Root Mean Square Error of Approximation (RMSEA), and Comparative Fit Index (CFI) were used. Specifically, a non-significant χ2 value indicates that the theoretical model is consistent with the data, while RMSEA < 0.06, and CFI > 0.90, indicate an acceptable fit [23]. However, these fit indices show that the three-factor model did not adequately fit the observed data.

Based on these results and the recommendations of Marsh and colleagues [24], we conducted Exploratory Factor Analysis (EFA) to identify a possible alternative model. Consistent with MacCallum and Austin's [25] perspective, our goal was to identify another parsimonious and substantively meaningful model that reports a measurement of the relationships between observed variables and latent factors reasonably well. As assumptions for conducting EFA, item distribution, the Kaiser-Mayer-Olkin (KMO) value, and Bartlett's test of sphericity were tested. The Kaiser-Mayer-Olkin (KMO) test is considered adequate when the index is > 0.6 [26]. Bartlett’s test of sphericity is considered adequate when the p value < 0.05. Furthermore, asymmetry and kurtosis were performed for each variable to demonstrate a normal item distribution. The EFA was performed using a maximum likelihood procedure. Maximum likelihood is the best option when the data are reasonably normally distributed since "it allows for the computation of a wide range of indexes of the goodness of fit of the model” [27]. Then, oblimin non-orthogonal factor rotation was applied because the extracted factors may be correlated. Factor loadings were set at a coefficient level greater than |0.30| [26]. To evaluate the model fit, the χ2, χ2/degrees of freedom (df) value, RMSEA and Tucker-Lewis Index (TLI) were considered. As said before, a non-significant χ2 value and RMSEA < 0.06 indicate that the theoretical model is consistent with the data. A χ2/df value of ≤ 5 and TLI values > 0.9 are indicative of adequate fit [28], [29]. To evaluate the factor’s structure the ‘parallel analysis’ method [30] was applied and the scree plot was reviewed. Then, correlations between factors were calculated.

#### Reliability

Once the factorial structure of the scale was analyzed, we performed further tests to verify the reliability of the instrument. To test internal consistency, we calculated Cronbach’s alpha for each subscale and the total scale. As an exploratory study introducing a new scale, a value of Cronbach’s alpha > 0.6 has been considered sufficient [31], [32], and alpha > 0.8 is desirable. To check repeatability, a one-month test-retest was performed; Pearson correlation was tested.

The IBM SPSS Statistics 28 and JAMOVI (version 2.3) were used to perform statistical analysis.

#### Ethics

The study was conducted in accordance with principles of the Declaration of Helsinki and its subsequent amendments. The study was approved by the Ethical Committee of a Scientific Institute for Research, Hospitalization and Healthcare in Italy. All patients provided informed consent prior to participation in the study (N. 243_2023bis / 24.04.2023).

## Results

### Sociodemographic and clinical data

The study sample comprised 414 women, with a mean ± SD age of 35.5 ± 4.62 years. Most participants (50.5 %) were married to the father of their child, 48.3 % were cohabiting, and 1.2 % were single or divorced. Of the total participants, 55.6 % were primiparous and 44.4 % were multiparous. Most of the women had spontaneous conception, while 12.1 % had conception through assisted reproduction technology treatment, and 1 % with the aid of pharmacological treatment. Regarding delivery, 56 % of the women had a natural delivery, 4.6 % had an assisted vaginal childbirth (i.e., forceps), and 39.4 % had a caesarean section. All sociodemographic and clinical characteristics of the study population are shown in Table 1.

**Table 1:** Descriptive statistics of the sample.

Sample size = 414		
	*N*	*%*
*Education*		
Middle school diploma	17	4,1
Professional course license	13	3,1
High school diploma	100	24,2
University degree	225	54,3
Postgraduate masters/PhD	53	12,8
Other	6	1,4
*Job title*		
Freelance professional	56	13,5
Employed	315	76,1
Unemployed	17	4,1
Housewife	12	2,9
Student	2	0,5
Other	12	2,9
*Pregnancy*		
Spontaneous fertilization	360	87,0
Pharmacological treatment	4	1,0
Assisted Reproductive Technology treatment	50	12,1
Single pregnancy	401	96,9
Twin pregnancy	13	3,1
Primiparity	230	55,6
*Complications*		
No	374	90,3
Haemorrhage	4	0,97
High pressure	6	1,45
Preeclampsia	2	0,48
*Previous miscarriage*		
No	274	66,2
Spontaneous miscarriage	98	23,7
Voluntary interruption of pregnancy	36	8,7
Therapeutic termination of pregnancy	6	1,4
*Childbirth*		
Natural childbirth	232	56,0
Operative Vaginal Delivery	19	4,6
Caesarean childbirth	163	39,4

### Factor analysis

The Preliminary tests were conducted prior to factor extraction to verify that the data satisfied the psychometric criteria for the factor analysis. Specifically, the KMO coefficient was “marvelous” (KMO=0.897), and Bartlett's test of sphericity was significant (χ2 = 6552.35, df = 528, p < 0.001). Asymmetry and kurtosis values of each variable confirmed multivariate normality. Thus, these findings indicate that the data satisfied the psychometric criteria for the factor analysis. CFA was performed to test the original three-factor theoretical model introduced by Ford and colleagues [17]. However, the CFA solution showed a poor fit: the theoretical model did not adequately fit the observed data. Specifically, χ2 = 2100, df= 492, p < 0.001, RMSEA = 0.0888 (0.085 – 0.0928), and CFI = 0.45. Factor loadings of some items were below 0.30 (for example, factor loading of item 5 “The people in the room took control” was = −0.013; factor loading of item 28 “I felt the staff had their own agenda” was = 0.179) and they were not significant at p < 0.001.

Hence, we decided to execute the EFA. Since the Bartlett Test of Sphericity (BTS) and Kaiser-Meyer-Olkin (KMO) measure of sample adequacy ensure that data were suitable, an EFA was conducted on the entire sample. EFA was performed using the maximum likelihood method with oblimin non-orthogonal factor rotation. EFA yielded a six-factor structure. The six factors were named: Support: healthcare professionals’ guidance; Support: healthcare professionals’ presence; External control: control over medical procedures; External control: control over the informational process; Internal control: control over emotional and physical reactions; Internal control: control over pain. These factors replicate those found by Ford and colleagues [17], each divided into two further subfactors.

Table 2 shows the six factors, corresponding items, and saturation of each item.

**Table 2:** Results of the exploratory factor analysis (N = 414).

	*Factor*					
*Items*	*1*	*2*	*3*	*4*	*5*	*6*
1. I had control over when procedures happened		0364				
2. I could influence which procedures were carried out		0895				
3. I decided whether procedures were carried out or not		0848				
4. I had control over the decisions that were made		0812				
5. The people in the room took control^r^.	0331					
6. People coming in and out of the room was beyond my control.						
7. I could get up and move around as much as I wanted.						
8. I chose whether I was given information or not				0796		
9. I could decide when I received information				0926		
10. I had control over what information I was given				0818		
11. I felt I had control over the way my baby was finally born		0468				
12. The pain was too great for me to gain control over it.^r^					0801	
13. I was overcome by the pain.^r^					0902	
14. I was mentally calm.			0739			
15. I was able to control my reactions to the pain			0770			
16. I was in control of my emotions.			0809			
17. I felt my body was on a mission that I could not control.						
18. Negative feelings overwhelmed me.^r^			0326			
19. I gained control by working with my body.			0601			
20. I behaved in a way not like myself.^r^						
21. I could control the sounds I was making.			0441			
22. The staff helped me find energy to continue when I wanted to give up	0804					
23. The staff seemed to know instinctively what I wanted or needed.	0811					
24. The staff went out of their way to try to keep me comfortable.	0759					
25. The staff encouraged me to try new ways of coping (such as breathing)	0800					
26. The staff encouraged me not to fight against what my body was doing.	0617					
27. The staff realized the pain I was in.	0657					
28. I felt the staff had their own agenda.^r^						0547
29. I was given time to ask questions.	0418					
30. I felt like the staff tried to move things along for their own convenience.^r^						0789
31. The staff helped me to try different positions.	0439					
32. The staff stopped doing something if I asked them to stop.	0357					
33. The staff dismissed things I said to them.^r^						0532

r Item is reverse scored; Absent values: factor loadings are less than 0.30.

Furthermore, on the basis of saturation, item 5 (“The people in the room took control”) was moved from the scale related to Internal Control to the scale related to Support, specifically Support: healthcare professionals’ guidance. This shift could be motivated by context, as the entry and exit of people during childbirth is strongly controlled by healthcare professionals. Since factor loadings were set at a coefficient level greater than |0.30|, we removed item 6 (“I felt my body was on a mission that I could not control”), item 10 (“I behaved in a way not like myself”), item 16 (“I could get up and move around as much as I wanted”), item 17 (“People coming in and out of the room was beyond my control”). The EFA solution showed a good fit: RMSEA = 0.0404 (0.035–0.046), and TLI = 0.941. Moreover, χ2 = 576, p < .001; however, the χ2/df value = 1.67; < 5. As the indices show the goodness of fit of the model, this theoretical framework can be applied to the Italian version of the SCIB.

Correlation between factors values ranges from 0.012 to 0.306. The correlation coefficient was sufficient low to maintain the division between them [27].

### Reliability

The overall Cronbach’s alpha of all 29 items in the I-SCIB was 0.82, showing good internal consistency. The Cronbach’s alpha values of the six factors were: External control: control over medical procedures (α = 0.89), External control: control over the information process (α = 0.88), Internal control: control over emotional and physical reactions (α = 0.60), Internal control: control over pain (α = 0.84), Support: healthcare professionals’ guidance (α =.75), and Support: healthcare professionals’ presence (α = 0.68). Cronbach’s alpha for all of the subscales of the original SCIB are reported for comparison: Cronbach’s alpha for the 33 items was 0.81; Cronbach’s alpha for Internal Control = 0.74; Cronbach’s alpha for External Control = 0.71; Support = 0.64.

The correlation value between the total scores of the two SCIB administrations over a month time period was r = 0.631, p < 0.001, indicating an acceptable temporal stability.

## Discussion

The SCIB scale is a validated instrument that assesses maternal perceptions of support and control during childbirth. Several studies have shown that high control and support might positively influence childbirth experiences [7], [8], [9], [10], [11]; however, tools for measuring control are often one-dimensional, focusing on single aspects of control - internal or external as well as single dimension of control (e.g., decision-making, control of pain, access to information, etc.). For example, Brand and colleagues [33] used the Perceived Control in Childbirth Scale and focused on mothers’ perceived autonomy; Baranowska and colleagues [34] used an ad hoc questionnaire and focused on the quality of information provided by the medical staff. Furthermore, in some studies control emerges as one of the important factors of the experience through a qualitative approach on small samples [35, [36], [37], [38].Thus, Ford and colleagues [17] developed the SCIB scale to introduce a measure of control (and support), as a multidimensional construct. This study aimed to introduce the Italian version of the SCIB and analyze its psychometric properties.

After the Italian translation of the SCIB through back-translation method, factor analysis was performed to explore the internal structure of the SCIB. Since the SCIB scale has already been validated in various cultures, construct validity was first analyzed using CFA. The CFA allowed the assessment of the validity of the latent constructs and testing of the hypothesized factor theoretical model. The original theoretical model was obtained through a multi-methodological research project by Ford and colleagues [17] which yielded a theoretical model with three dimensions: internal control, external control, and support. In our study, CFA was conducted to test whether this theoretical model could also be applied in the Italian context. However, the fit indices of our CFA showed a poor adjustment. It could be hypothesized that the model misfit may be attributable to several factors. First, broader issues of cultural and linguistic adaptation must be considered. For example, some items, such as “The people in the room took control” and “People coming in and out of the room was beyond my control” may be ambiguous in the Italian context, as they do not reflect the logistical norms of local hospitals and could have led to differences in interpretation. Second, some items did not achieve a factor loading greater than 0.30, suggesting that they did not adequately represent the latent constructs. Finally, the homogeneity of the sample may have limited the variance needed to confirm the original structure. Although the professional group included several healthcare professionals, participants were recruited from a single institution. It is important to check whether further studies with different recruitment strategies might produce different results.

Hence, EFA was performed to identify another parsimonious and substantively meaningful model that reported a measurement of the relationships between observed variables and latent factors reasonably well [25]. EFA yielded a six-factor model, named “Support: healthcare professionals’ guidance”, “Support: healthcare professionals’ presence”, “External control: control over medical procedures”, “External control: control over the informational process”, “Internal control: control over emotional and physical reactions”, and “Internal control: control over pain.”. The EFA results are consistent with the theoretical model of Ford and colleagues [17]: the six factors, indeed, are closely aligned to those originally proposed and appear to decline them at a more specific level. Regarding external control, our findings specified the object of control, distinguishing between control of the information process (item 19: “I could decide when I received information”) or control of medical procedures (item 13: “I decided whether procedures were carried out or not”). Similarly, for internal control, the data distinguished between control over emotions and behaviors (item 5 “I was in control of my emotions”) or pain (item 1 “The pain was too great for me to gain control over it”). Finally, a distinction can be made between support in the form of guidance of the healthcare professionals during childbirth (item 22 “The staff helped me find energy to continue when I wanted to give up”) or support in the form of presence/absence of healthcare professionals (item 28 “I felt the staff had their own agenda”). The identified factors also reflect the sub-themes that emerged from the interview analysis conducted by Ford and colleagues [17]. Moreover, there was a correlation between some of the six factors, but the correlation coefficient was sufficiently low to maintain the division between them [24].

Assessing factor loading, item 5 was moved to another factor. Furthermore, the EFA showed that four items did not have appropriate factor loading, so item 6, item 7, item 17, and item 20 were removed. These changes were probably due to context-specificity. Indeed, 'People coming in and out of the room was beyond my control,' could be interpreted differently in the Italian context since the presence during childbirth is strictly reserved for the partner and managed by healthcare professionals. Thus, the Italian version of the SCIB consists of 29 items.

The fit indices showed that the six-factor structure has good evidence of validity. It is noteworthy that the Italian SCIB also has acceptable reliability. Specifically, the scale showed acceptable temporal stability and good internal consistency.

Despite these potentialities, our study has some limitations. First, data were collected using a convenience sample of women enrolled in the same postnatal ward. Although recruitment took place in one of the largest Italian hospitals, the fact that all participants come from a single hospital means that they interacted with a relatively narrow group of maternity care professionals during the perinatal period. Furthermore, women with current diagnosis of severe mental health problems as well as those with serious pregnancy or birth complications were excluded. Therefore, our results may not generalize to these important groups, who could be at highest risk of poor psychological outcomes following a difficult childbirth. Future studies could carry out replications and further confirm the reliability of SCIB. Second, the primary objective was to validate the SCIB scale; therefore, limited attention has been paid to exploring the quality of experience of women. Future studies should adopt different samples and diverse recruitment strategies in order to confirm these results. Moreover, further contributions should explore the role of support and control in relation to outcomes such as individual psychological and relational well-being. Although the experience of labour and birth has been increasingly investigated by scientific literature, to date less attention was paid on the role of healthcare professionals in shaping the quality of this experience. More knowledge could lead to better care for women during their birthing experience.

This study contributes to knowledge about childbirth by cross-culturally adapting an instrument to measure perceptions of control and support during the birth experience. SCIB could help health professionals to monitor women's childbirth experiences and introduce tailored interventions when needed. In light of this study’s results, future studies should conduct confirmatory factor analysis to confirm the 6-factor structure. Furthermore, it would be important to assess the role of control and support in shaping the quality of the birth experience.

## Conclusions

This study showed that the SCIB could be translated to the Italian language and performed similarly in the Italian context to the UK context where it was delivered. Some items did not translate culturally and were moved between subscales or removed, but overall, the underlying factor structure was retained, with some additional consideration of the types of care that made women feel in control and supported. This new Italian version of the SCIB can be taken forward to measure Italian-speaking women’s birth experiences and link their perceptions of support and control to psychological and physical outcomes of childbirth.

## CRediT authorship contribution statement

**Sara Molgora:** Writing – review & editing, Writing – original draft, Validation, Supervision, Resources, Project administration, Methodology, Investigation, Formal analysis, Data curation, Conceptualization. **Federica Bonazza:** Writing – review & editing, Writing – original draft, Validation, Methodology, Investigation, Formal analysis, Data curation. **Maurizio Barbieri Carones:** Investigation, Data curation. **Enrico Maria Ferrazzi:** Investigation, Data curation. **Elizabeth Ford:** Writing – review & editing.

## Funding

This research did not receive any specific grant from funding agencies in the public, commercial, or not-for-profit sectors.

## Declaration of Competing Interest

The authors declared no potential conflicts of interest with respect to the research, authorship, and/or publication of this article.
